# miR-378 affects metabolic disturbances in the *mdx* model of Duchenne muscular dystrophy

**DOI:** 10.1038/s41598-022-07868-z

**Published:** 2022-03-10

**Authors:** Paulina Podkalicka, Olga Mucha, Katarzyna Kaziród, Krzysztof Szade, Jacek Stępniewski, Liudmyla Ivanishchuk, Hirofumi Hirao, Ewelina Pośpiech, Alicja Józkowicz, Jerzy W. Kupiec-Weglinski, Józef Dulak, Agnieszka Łoboda

**Affiliations:** 1 Department of Medical Biotechnology, Faculty of Biochemistry, Biophysics and Biotechnology, Jagiellonian University in Krakow, 30-387 Kraków, Poland; 2 The Dumont-UCLA Transplantation Center, Department of Surgery, Division of Liver and Pancreas Transplantation, David Geffen School of Medicine at UCLA, Los Angeles, CA 90095 USA; 3 Malopolska Centre of Biotechnology, Jagiellonian University in Krakow, 30-387 Kraków, Poland

**Keywords:** Diseases, Biochemistry, Carbohydrates, Lipids, Molecular biology, Non-coding RNAs, Transcriptomics

## Abstract

Although Duchenne muscular dystrophy (DMD) primarily affects muscle tissues, the alterations to systemic metabolism manifested in DMD patients contribute to the severe phenotype of this fatal disorder. We propose that microRNA-378a (miR-378) alters carbohydrate and lipid metabolism in dystrophic *mdx* mice. In our study, we utilized double knockout animals which lacked both dystrophin and miR-378 (*mdx*/miR-378^−/−^). RNA sequencing of the liver identified 561 and 194 differentially expressed genes that distinguished *mdx* versus wild-type (WT) and *mdx*/miR-378^−/−^ versus *mdx* counterparts, respectively. Bioinformatics analysis predicted, among others, carbohydrate metabolism disorder in dystrophic mice, as functionally proven by impaired glucose tolerance and insulin sensitivity. The lack of miR-378 in *mdx* animals mitigated those effects with a faster glucose clearance in a glucose tolerance test (GTT) and normalization of liver glycogen levels. The absence of miR-378 also restored the expression of genes regulating lipid homeostasis, such as *Acly*, *Fasn*, *Gpam*, *Pnpla3*, and *Scd1*. In conclusion, we report for the first time that miR-378 loss results in increased systemic metabolism of *mdx* mice. Together with our previous finding, demonstrating alleviation of the muscle-related symptoms of DMD, we propose that the inhibition of miR-378 may represent a new strategy to attenuate the multifaceted symptoms of DMD.

## Introduction

The lack of functional dystrophin is the primary cause of Duchenne muscular dystrophy (DMD) and is responsible for irreversible muscle weakening, with cardio-respiratory failure being the most common cause of premature death. However, the secondary consequences of dystrophin loss, though much less understood, significantly contribute to the severity of the disease. Accordingly, DMD should be recognized as a multi-systemic disorder and interdisciplinary care is recommended for the optimal management of DMD-associated complications^1^.

Disturbances in angiogenesis^2,3^ and brain^4^, renal, and bladder dysfunctions^5,6^, as well as bone-health issues^7^ and gastrointestinal problems^8^, reflect the range of pathological changes observed in DMD. Finally, systemic alterations in the metabolism of carbohydrates and lipids accompanied by cases of impaired glucose tolerance and insulin sensitivity are manifested by DMD patients and mouse models of the disease, including *mdx* mice and golden retriever muscular dystrophy (GRMD) dogs^9–13^.

Though a few mutation-specific compounds are used in clinical settings and other cell and gene therapy-based approaches are under extensive investigation, drugs that act mostly as anti-inflammatory agents, such as glucocorticoids (GCs), still serve as the gold standard of care for all patients suffering from DMD^14^. Apart from their indisputable beneficial effects in prolonging ambulation and improving the quality of the patient’s life, they, unfortunately, entail a list of multi-organ side effects^15^. This emphasizes the persistent need to evaluate novel targets to ameliorate disease symptoms, which could also potentially be applicable in the emerging concept of combined therapies for DMD patients^16^.

Given all of the above, factors that influence both muscular and systemic perturbations might be of special interest. One candidate might be short, noncoding microRNAs (miRs), due to their potential to modulate a wide range of physiological and pathological processes^17^. We have recently studied miR-378a (miR-378), embedded in the first intron of the peroxisome proliferator-activated receptor-gamma coactivator 1β (*Ppargc1b*) gene encoding PGC1β, a factor that has been implicated in energy metabolism^18^. miR-378 plays a multifaceted role in metabolism, angiogenesis, and muscle biology^18^. In the context of the latter, we and others have demonstrated that miR-378 is abundantly expressed in skeletal and cardiac tissues^19,20^ and that its deficiency influences vascularization of skeletal muscles^19^ as well as the functional properties and size of cardiomyocytes^21^, whereas overexpression of miR-378 attenuates muscle regeneration^22^. Notably, we previously demonstrated that the lack of miR-378 mitigates muscle dystrophy by reducing inflammation, decreasing fibrosis, and affecting the properties of satellite cells, the *bona fide* muscle stem cells. Most importantly, this was associated with increased physical performance and enhanced contractile properties of a single muscle. Surprisingly, RNA sequencing (RNA-seq) analysis of the gastrocnemius muscle revealed no profound transcriptional changes between *mdx*/miR-378^−/−^ and *mdx* mice. On the other hand, the analysis of muscle fiber type composition pointed toward the influence of miR-378 on metabolism^23^. Accordingly, as miR-378 has been recognized as a potent modulator of lipid and carbohydrate homeostasis^19^ and was proposed as a biomarker of insulin resistance^24^, we examined whether the higher muscle functionality of *mdx* mice lacking miR-378 might be accompanied by improved systemic metabolism. Carrer et al. demonstrated previously that the loss of miR-378 diminished liver fat deposition while facilitating the response to glucose administration during a glucose tolerance test (GTT) in mice fed a high-fat diet^25^. Others reported that miR-378 regulates hepatic insulin signaling by targeting the p110α catalytic subunit of PI3K^26,27^, providing evidence for a hepatic-related function of miR-378. Though the incidence of hepatitis^28^, non-alcoholic fatty liver disease^29^, drug-induced hepatotoxicity^30^, or even acute liver failure secondary to dilated cardiomyopathy^31^ have been observed in DMD patients, these hepatic alterations are not well understood. Murphy et al.^32^ evaluated the liver proteome of the *mdx*-4Cv model of DMD and found dysregulated levels of the factors implicated in fatty acid and carbohydrate metabolism, among other findings. However, no studies have examined any candidate factors that would mediate those effects.

Higher levels of miR-378 in the serum/plasma of dystrophic animals and DMD patients, most likely a result of its leaking from damaged muscles, was reported previously by us^23^ and others^33^. Circulating miRNAs are often bound to lipoproteins, especially high-density lipoprotein (HDL) and low-density lipoprotein (LDL) cholesterol. In such complexes, they might be delivered to the liver^34^, which could also be the case regarding miR-378. In light of the above, in the current study, we sought to uncover liver pathology driven by dystrophin loss in an *mdx* model of DMD and to verify the hypothesis that a loss of miR-378 improves disturbed carbohydrate and lipid homeostasis in dystrophic animals. This, together with our previous findings^23^, sheds light on how the absence of miR-378 improves the dystrophic phenotype.

## Results

### RNA-seq revealed notable alterations in the hepatic transcriptome of *mdx* and *mdx*/miR-378^−***/***−^ mice

**Figure 1:**
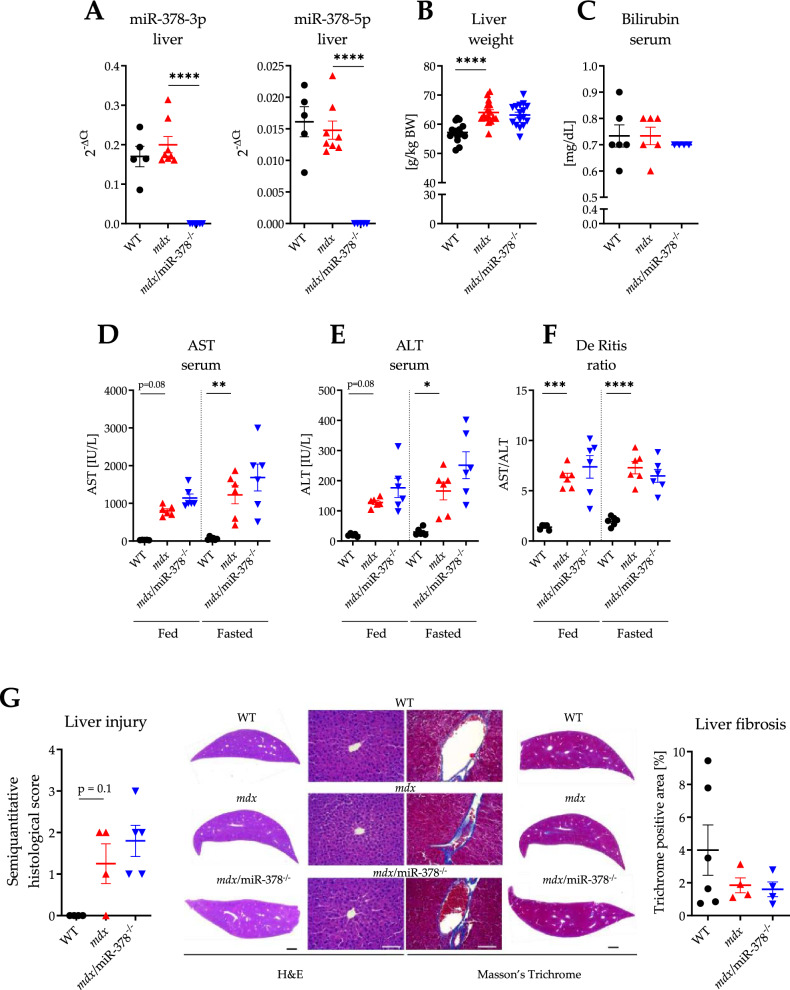
The global lack of miR-378 in *mdx* mice (*mdx*/miR-378^−/−^) does not profoundly affect liver histology and injury markers. (**A**) The level of both mature strands of miR-378, namely miR-378-3p and miR-378-5p assessed with the use of locked nucleic acid (LNA) technology; n = 5–8/group. (**B**) The liver weight was presented as g/kg BW; n = 15–16/group. (**C**) Bilirubin concentration determined in the serum by biochemical analyzer SPOTCHEM; n = 14–15/group. The level of (**D**) aspartate transaminase (AST) and (**E**) alanine transaminase (ALT) together with (**F**) calculated De Ritis ratio of AST/ALT in the serum collected from mice under non-fasting conditions (fed) and after overnight fasting (fasted); measurements performed with the use of biochemical analyzer SPOTCHEM; n = 5–6/group. (**G**) Liver histology was assessed by hematoxylin and eosin (H&E) and Masson’s Trichrome staining on paraffin-embedded sections, visualized with the use of Leica DMi8 microscope with CMOS Leica MC170 HD camera, and analyzed semiquantitatively (H&E) and quantitatively (Masson’s Trichrome); n = 4–6/group. Scale bars represent 1 mm (black) and 100 μm (white). Data are presented as mean ± SEM. *p < 0.05; **p < 0.01; ***p < 0.001; ****p < 0.0001 by one-way ANOVA with Tukey’s post-hoc test.

Undetectable levels of mature strands of miR-378, miR-378-3p, and miR-378-5p, were confirmed in both the liver (Fig. 1A) and isolated hepatocytes (Supplementary Fig. 1A) of *mdx*/miR-378^−/−^ mice, along with no apparent differences in miR-378 expression between the *mdx* and WT cohorts.

Higher relative liver weight was noticed in dystrophic mice without further changes driven by miR-378 loss (Fig. 1B), whereas the serum level of a typical marker of liver injury, bilirubin, did not differ between genotypes (Fig. 1C). On the contrary, both aspartate aminotransferase (AST, Fig. 1D) and alanine aminotransferase (ALT, Fig. 1E), whose levels increase as a result of both liver and muscle damage—as in the case of DMD—were markedly elevated in the *mdx* mice under normal fed conditions and after fasting overnight. This was also reflected by the substantially higher de Ritis ratio, which was not further escalated in the *mdx*/miR-378^−/−^ mice (Fig. 1F).

**Figure 2:**
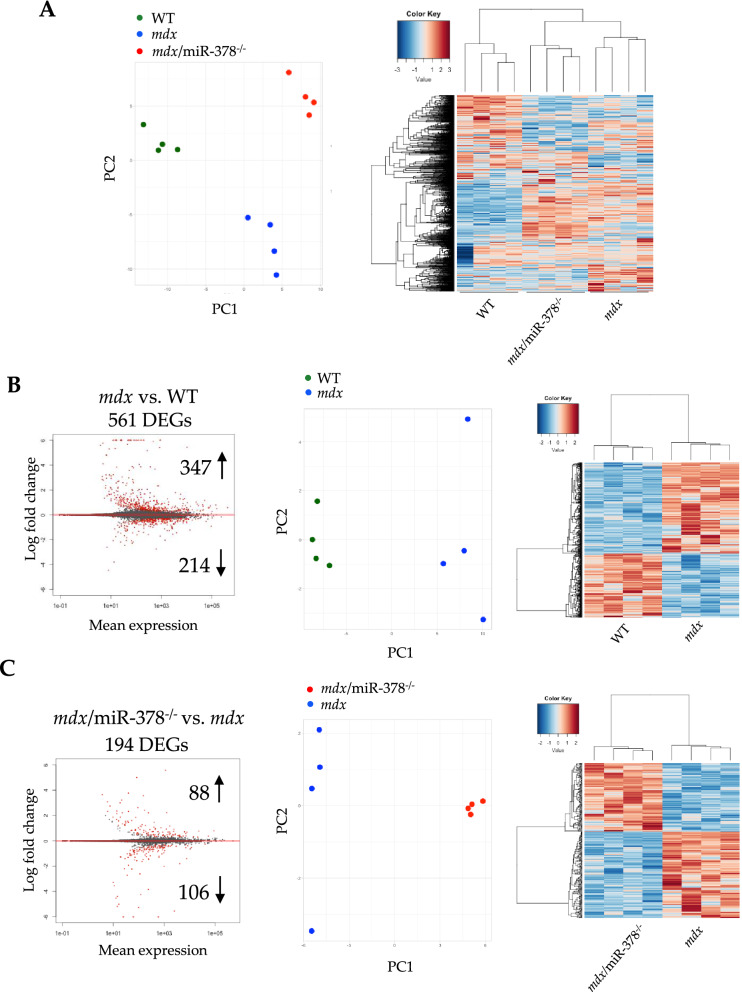
Alterations in the liver transcriptome of *mdx* mice are affected by the additional lack of miR-378. (**A**) Transcriptome profiling by RNA-seq in the *mdx* vs. WT mice and mice lacking both dystrophin and miR-378 (*mdx*/miR-378^−/−^) vs. *mdx* segregates the investigated groups into 3 distinct clusters based on 1000 most variable genes, n = 4/group. (**B**) RNA-seq analysis revealed 561 DEGs between *mdx* vs. WT livers, and (**C**) 194 DEGs between *mdx*/miR-378^−/−^ vs. *mdx* that distinguish the analyzed groups by principal component analysis (PCA) and hierarchical clustering.

Simultaneously, the histological assessment did not reveal severe liver injury, collagen deposition abnormalities (Fig. 1G), or lipid deposition (Supplementary Fig. 2) in any of the cohorts. Despite that, RNA-seq analysis showed profound changes in the liver transcriptome. The 1000 most variable genes separated the WT, *mdx,* and *mdx/*miR-378^−/−^ mice by the first and second principal components (PC1 and PC2, respectively) (Fig. 2A). Accordingly, the *mdx* and *mdx/*miR-378^−/−^ mice clustered together, while the WT mice formed the second main cluster, indicating that the lack of dystrophin had the strongest impact on the gene expression profile in the liver (Fig. 2A). Of note, DESeq2 analysis identified 561 differentially expressed genes (DEGs) that distinguished *mdx* against WT and 194 DEGs that differentiated *mdx*/miR-378^−/−^ from *mdx* livers (Fig. 2B, C), by hierarchical clustering and principal component analysis (PCA).

**Figure 3:**
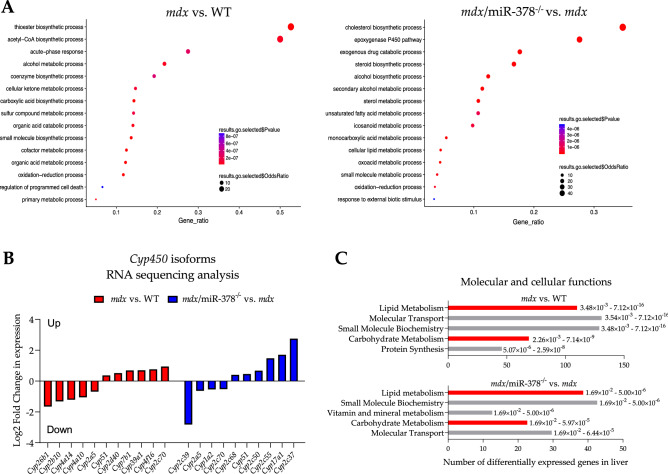
Metabolic pathways are predominantly changed in the livers of both *mdx* and *mdx*/miR-378^−/−^ mice. (**A**) Analysis of gene ontology (GO) terms of affected pathways. (**B**) The graph showing higher and lower expression of cytochrome P450 (CYP450) isoforms based on RNA-seq results, presented as the fold change. (**C**) Affected pathways associated with molecular and cellular functions were determined by ingenuity pathway analysis (IPA) and arranged according to the *p*-value range.

Gene ontology (GO) analysis pointed toward metabolism-related pathways, which were placed among the most significantly affected GO terms (Fig. 3A). Reduced basal respiration of dystrophic hepatocytes was revealed, without any apparent effect of miR-378 loss (Supplementary Fig. 1B), which is in line with the metabolic impairment observed in dystrophic liver mitochondria^35^. Moreover, the altered expression of cytochrome P450 (CYP450) enzymes—key players in xenobiotic and steroid metabolism^36^—was observed as a result of dystrophin deficiency and in *mdx/*miR-378^−^^*/*^^−^ mice (Fig. 3B). The molecular and cellular functions identified by Ingenuity Pathway Analysis (IPA) also confirmed that DEGs were associated with metabolic processes, particularly related to lipids and carbohydrates (Fig. 3C).

### The impaired glucose tolerance and insulin sensitivity displayed by *mdx* mice are affected by the loss of miR-378

**Figure 4:**
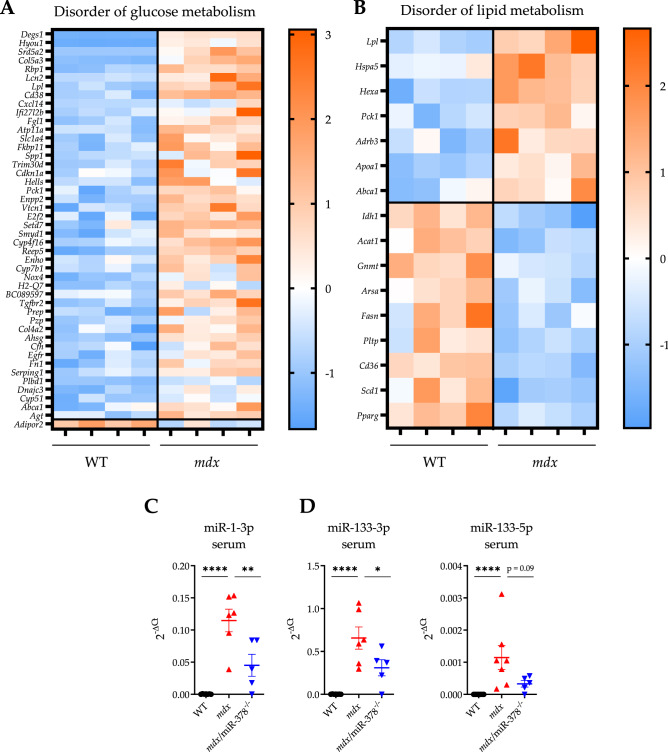
Disorder of glucose and lipid metabolism in the *mdx* mice is accompanied by higher expression of miR-1 and miR-133, further diminished as the result of the miR-378 loss. (**A**, **B**) Heat maps were created based on the list of genes indicated by ingenuity pathway analysis (IPA) that were attributed to the disorder of glucose (*p*-value = 1.04 × 10^–12^) and lipid metabolism (*p*-value = 1.31 × 10^–6^) in the liver of *mdx* mice. Blue color indicates downregulation, whereas orange upregulation. The expression of (**C**) miR-1-3p, (**D**) miR-133-3p, and miR-133-5p in the serum as assessed by locked nucleic acid (LNA) technology; n = 5–7/group. Data are presented as mean ± SEM. *p < 0.05; **p < 0.01; ****p < 0.0001 by one-way ANOVA with Tukey’s post-hoc test.

Further analysis revealed that the vast majority of genes regulating glucose metabolism (Fig. 4A) were elevated in the dystrophic mice (except for *Adipor2*), whereas factors related to lipid metabolism disorder displayed both upregulated and downregulated patterns (Fig. 4B). Additionally, the serum profile of miRNAs known predominantly as muscle-specific miRs (myomiR)^37^—but also reported as potential biomarkers of pre-diabetic/diabetic states and associated with glucose intolerance and insulin resistance^38,39^—was altered in the *mdx* and *mdx*/miR-378^−/−^ animals. Accordingly, miR-1 (Fig. 4C) and miR-133 (Fig. 4D) levels were elevated in the *mdx* mice in comparison with the WT mice and were lower as a result of the additional lack of miR-378.

**Figure 5:**
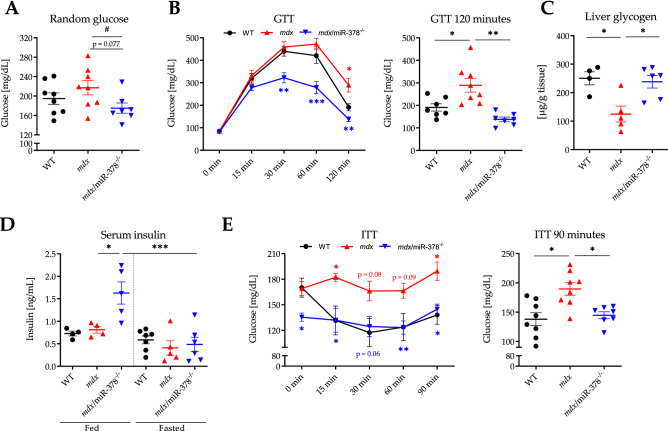
Disturbed systemic glucose homeostasis in the *mdx* mice is affected by the miR-378 loss. (**A**) The glucose level under non-fasting conditions was measured with a glucometer in the blood from the tail tip; n = 7–8/group. (**B**) A glucose tolerance test (GTT) was performed after overnight fasting. Glucose concentration was assessed in the blood from the tail tip before (0 min) and 15, 30, 60, and 120 min after i.p. injection of glucose solution; n = 7–8/group. (**C**) Liver glycogen content was evaluated after overnight fasting with the use of a biochemical assay; n = 4–6/group. (**D**) Serum insulin concentration was determined both under non-fasting conditions (fed) and after overnight fasting (fasted) by ELISA; n = 4–7/group. (**E**) Insulin tolerance test (ITT) was performed after 4 h of fasting. Glucose concentration was measured in the blood from the tail tip before (0 min) and 15, 30, 60, 90 min after intraperitoneal injection of insulin; n = 7–8/group. Data are presented as mean ± SEM. *p < 0.05; **p < 0.01; ***p < 0.001 by (**A, C, D**) one-way ANOVA with Tukey’s post-hoc test or (**B, E**) by two-way ANOVA for repeated measures with Tukey’s post-hoc test. (**A**) ^#^*p* < 0.05 additionally tested by Student’s t-test for comparison of *mdx*/miR-378^−/−^ vs. *mdx* only.

To further study the possible role of miR-378 in disturbed carbohydrate homeostasis in dystrophic mice, we utilized the functional GTT and the insulin tolerance test (ITT). A slightly lower glucose level was observed among the *mdx*/miR-378^−/−^ mice even under fed conditions (random glucose), without apparent changes in the dystrophic animals (Fig. 5A). When the mice underwent a GTT, mild glucose intolerance was found in the *mdx* individuals, as indicated by an elevated glucose level 120 min after i.p. injection of glucose (Fig. 5B). Importantly, the *mdx*/miR-378^−/−^ mice rapidly cleared the glucose, since diminished concentrations were visible even 30 min after injection and were sustained at a lower level than in the dystrophic animals in each subsequent time-point (Fig. 5B). This was accompanied by markedly reduced glycogen content in the *mdx* livers than in WT livers, restored to the level of the WT groups in *mdx*/miR-378^−^^/^^−^ mice (Fig. 5C). Additionally, the lack of miR-378 decreased the level of hepatic interleukin 6 (IL6, Supplementary Fig. 3A), tumor necrosis factor (TNFα, Supplementary Fig. 3B), and its receptor (TNFR1, Supplementary Fig. 3C); these factors are positively correlated with glycogen degradation and insulin resistance^40–42^.

Insulin concentration in the serum was higher in the *mdx*/miR-378^−/−^ mice under fed conditions with no changes between the groups after overnight fasting (Fig. 5D). The dystrophic animals failed to respond to insulin in the ITT test, which resulted in markedly higher glucose levels in comparison to the WT mice 90 min after insulin administration, suggesting impaired insulin sensitivity in those animals (Fig. 5E). On the contrary, glucose concentration in the *mdx*/miR-378^−/−^ animals was already at a low level before insulin injection; thus, the further drop following insulin administration was rather subtle (Fig. 5E). Starting from 15 min, the glucose concentration was similar to that of the WT mice, remaining markedly lower in comparison to the dystrophic animals following the next measurements (Fig. 5E). Taking all of the above into consideration, we suspected an altered expression of glucose transporter type 4 (GLUT4) in the muscles as the cause of impaired glucose uptake from the bloodstream. Lower levels of *Glut4* mRNA were observed in the *mdx* animals as compared to the WT ones with no effect of miR-378 loss (Supplementary Fig. 4A). At the protein level, no alterations between genotypes were noticed in Western Blot (Supplementary Fig. 4B), whereas immunofluorescent staining revealed the presence of cytoplasmic aggregates of GLUT4 in the *mdx* animals and not the WT ones, without further changes resulting from an additional lack of miR-378 (Supplementary Fig. 4C).

### Complex regulation of factors influencing glucose and lipid homeostasis in the liver of *mdx* and *mdx*/miR-378^−***/***−^ mice

Given that miR-378 was already reported to affect energy metabolism, for example, by targeting p110α^25–27^ and insulin-like growth factor 1 receptor (IGF1R)^20^, we aimed to check the levels of those markers in our experimental settings, together with glycogen synthase kinase 3 beta (GSK-3β) and pAKT/AKT kinase, due to their well-established role in glucose homeostasis and insulin action^43^. Nonetheless, the hepatic level of p110α was even lower in the *mdx*/miR-378^−/−^ mice (Supplementary Fig. 5A,B). GSK-3β appeared to be potently upregulated in the *mdx* animals, without any further changes driven by the lack of miR-378 (Supplementary Fig. 5A,C), whereas IGF1R (Supplementary Fig. 5D) and pAKT/AKT (Supplementary Fig. 5E) displayed no differences between genotypes. We also analyzed the level of peroxisome proliferator-activated receptor alpha (PPARα), which plays a role not only in carbohydrate homeostasis^44,45^ but predominantly in fatty acid metabolism as well^46^. Despite the lack of changes in mRNA (Supplementary Fig. 5F), strong upregulation in the hepatic protein level of PPARα in the dystrophic mice with a concomitant decrease caused by miR-378 deficiency (Supplementary Fig. 5G) indicated altered lipid homeostasis.

### Factors related to lipid homeostasis as the candidate mediators of miR-378 action in the liver

**Figure 6:**
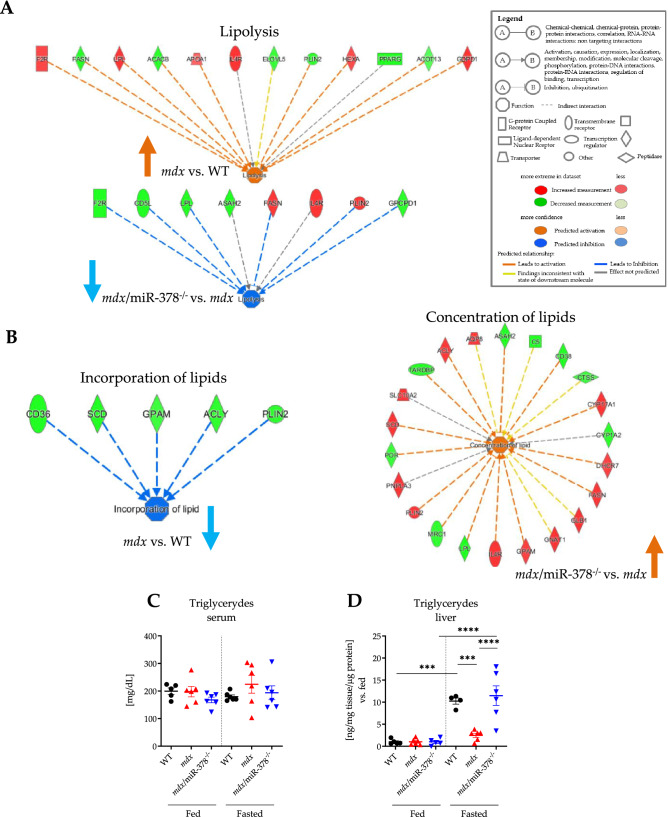
Disturbances in lipid metabolism in the liver of the *mdx* mice are affected by the additional lack of miR-378. (**A**) Ingenuity pathway analysis (IPA) revealed increased and decreased lipolysis in *mdx* vs. WT (*p*-value = 1.54 × 10^–4^) and *mdx*/miR-378^−/−^ vs. *mdx* (*p*-value = 1.28 × 10^−5^) livers, respectively. (**B**) Incorporation of lipids was shown to be lower in the liver of *mdx* mice (*p*-value = 6.33 × 10^–5^) whereas the concentration of lipids was higher in *mdx*/miR-378^−/−^ vs. *mdx* livers (*p*-value = 6.44 × 10^–5^). Triglyceride levels were assessed under non-fasting (fed) conditions and after overnight fasting (fasted) in (**C**) the serum; n = 5–6/group and (**D**) in the liver; 4–6/group. Data are presented as mean ± SEM. ***p < 0.001; ****p < 0.0001 by one-way ANOVA with Tukey’s post-hoc test.

The IPA-based analysis predicted the opposite regulation of lipolysis in *mdx* and *mdx*/miR-378^−/−^ livers – upregulation in the dystrophic animals and downregulation in the mice which additionally lacked miR-378 (Fig. 6A). Simultaneously, the incorporation of lipids was lower in the *mdx* mice, whereas the concentration of lipids was elevated in the *mdx*/miR-378^−/−^ animals (Fig. 6B). Since triglycerides are hydrolyzed in the process of lipolysis, we aimed to measure their levels in the serum and liver. Despite the lack of changes in the serum (Fig. 6C), hepatic triglycerides appeared to accumulate under fasting conditions in the WT and *mdx*/miR-378^−/−^ mice, which was not observed in the case of the dystrophic animals (Fig. 6D). No increase was noticed in the serum levels of total cholesterol (Supplementary Fig. 6A) and HDL (Supplementary Fig. 6B), under either fed or fasting conditions in any of the groups.

**Figure 7:**
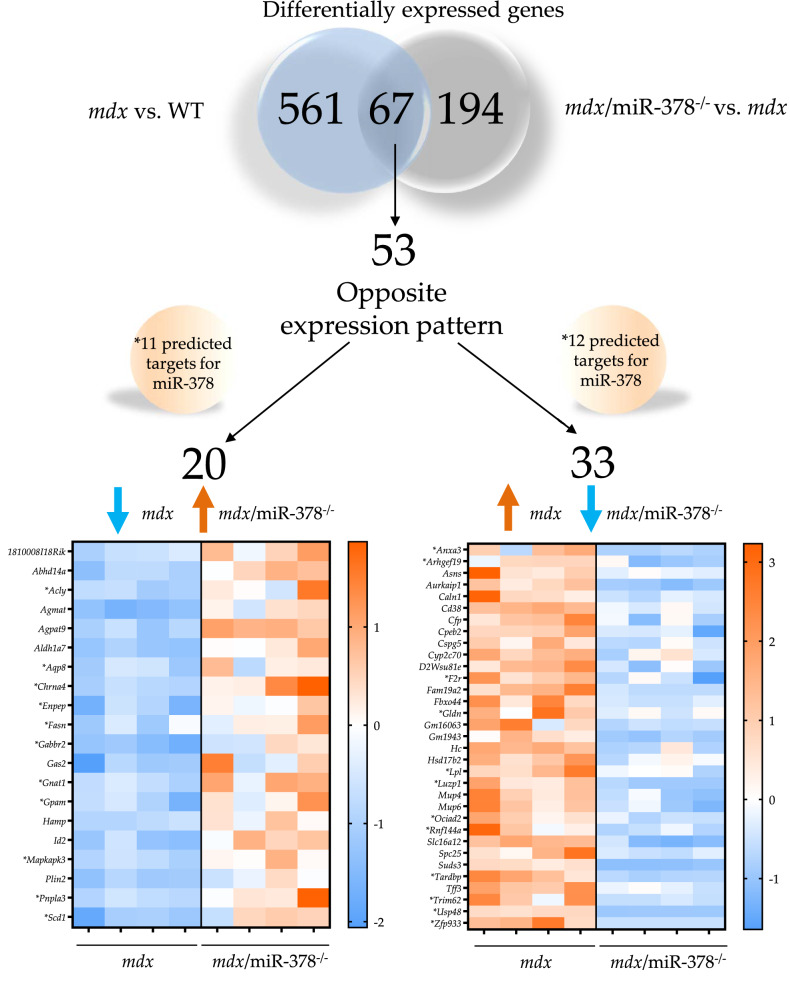
The analysis of oppositely expressed genes in the liver of the *mdx* vs. WT and *mdx*/miR-378^−/−^ vs. *mdx* mice. Based on RNA-seq analysis, 67 genes were found to be differentially expressed in both comparisons. 53 of them exhibited opposite expression patterns: 20 genes were downregulated in *mdx* and upregulated in *mdx*/miR-378^−/−^ livers, whereas 33 were elevated in *mdx* and downregulated in *mdx*/miR-378^−/−^ livers. The expression pattern and gene names are presented on heat maps. Orange color indicates upregulation whereas blue color downregulation. Asterisks indicate genes predicted to be miR-378 targets according to the miRmap database.

**Figure 8:**
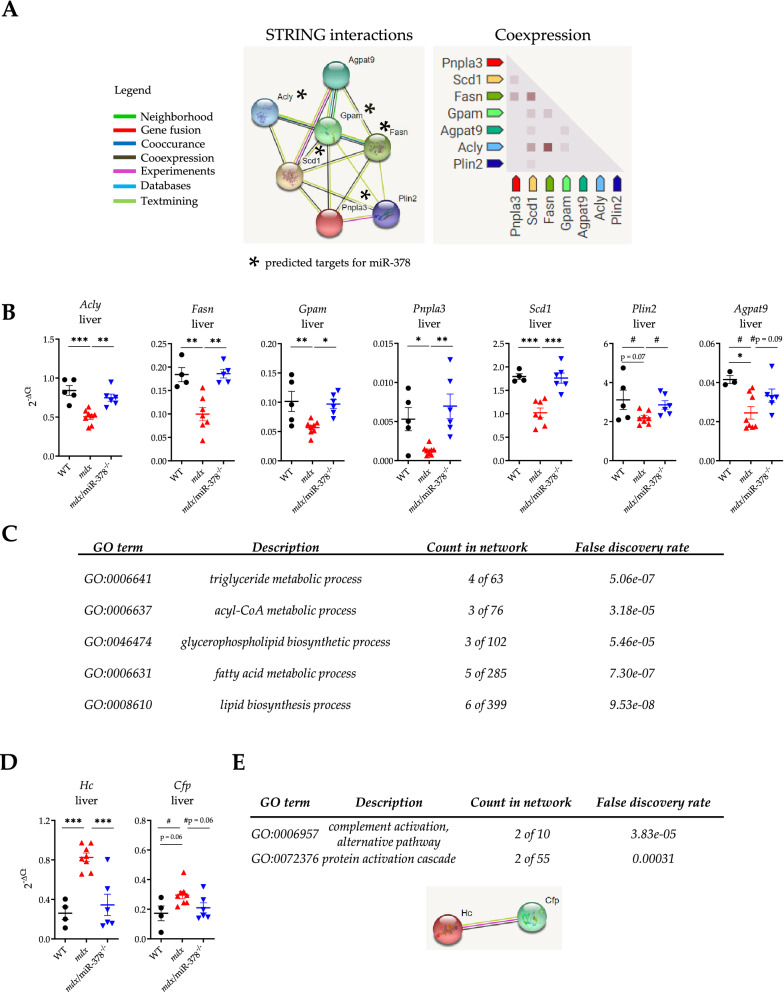
Search Tool for the Retrieval of Interacting Genes/Proteins (STRING)—based analysis uncovered possible mediators of miR-378 action in the liver. The STRING analysis was performed on differentially expressed genes that were oppositely regulated in *mdx* vs. WT and *mdx*/miR-378^−/−^ vs. *mdx* livers. (**A**) 7 out of 20 genes that were downregulated in *mdx* vs. WT and upregulated in *mdx*/miR-378^−/−^ vs. *mdx* mice were found by STRING to interact with each other and exhibit co-expression patterns. Five of them indicated by asterisks were predicted by miRmap database to be direct targets of miR-378. (**B**) Verification of RNA sequencing results by qRT-PCR analysis of ATP citrate synthase (*Acly*), fatty acid synthase (*Fasn*), glycerol-3-phosphate acyltransferase, mitochondrial (*Gpam*), patatin-like phospholipase domain-containing protein 3 (*Pnpla3*), and stearoyl-CoA desaturase-1 (*Scd1*) predicted as miR-378 targets along with perilipin 2 (*Plin2*) and glycerol-3-phosphate acyltransferase 3 (*Agpat9*); n = 4–8/group, together with (**C**) gene ontology (GO) terms indicating pathways affected by interacting genes. (**D**) The expression of 2 out of 22 genes, hemolytic complement (*Hc*) and complement factor properdin (*Cfp*), that were higher in *mdx* vs. WT and lower in *mdx*/miR-378^−/−^ vs. *mdx* liver based on RNA-seq data, was verified by qRT-PCR analysis; n = 4–8/group. (**E**) Those genes, presented together with the affected GO terms, were shown by STRING to interact with each other. Data are presented as mean ± SEM. *p < 0.05; **p < 0.01; ***p < 0.001 by one-way ANOVA with Tukey’s post-hoc test; ^#^*p* < 0.05 additionally tested by Student’s t-test for comparison of *mdx* vs. WT and *mdx*/miR-378^−/−^ vs. *mdx* only.

To indicate potential mediators of miR-378 action in the liver, we examined the expression pattern of all 561 DEGs in *mdx* vs. WT and 194 DEGs in *mdx*/miR-378^−/−^ vs. *mdx* mice (Fig. 7). Among them, 67 DEGs were identified as being changed in both comparisons, with 53 DEGs exhibiting the opposite expression pattern (Fig. 7). Accordingly, 20 of these DEGs had lower expression in *mdx* vs. WT mice and concomitantly higher expression in the *mdx*/miR-378^−/−^ vs. *mdx* animals, pointing toward those factors being directly regulated by miR-378. More than half of them (11 out of 20) were found to be predicted targets of miR-378 with the miRmap database^47^ (Fig. 7). On the other hand, an indirect regulation might be proposed in the case of 33 DEGs which had higher expression in *mdx* vs. WT and lower expression in *mdx*/miR-378^−/−^ vs. *mdx* livers, with 12 DEGs being indicated as predicted targets of miR-378 (Fig. 7). When we analyzed 20 DEGs that were downregulated in *mdx* in comparison with WT and were upregulated in *mdx*/miR-378^−/−^ over *mdx* using the Search Tool for the Retrieval of Interacting Genes/Proteins (STRING), 7 of them were found to have multi-level interaction (Fig. 8A). We further validated the lower level of all of these DEGs in the *mdx* when compared with the WT mice and higher expression in *mdx*/miR-378^−/−^ than the *mdx* animals (though to a lesser extent in the case of perilipin 2; *Plin2* and glycerol-3-phosphate acyltransferase 3; *Agpat9*) by qRT-PCR analysis (Fig. 8B). Five out of 7 DEGs were identified as predicted targets of miR-378: ATP citrate synthase (*Acly*), glycerol-3-phosphate acyltransferase, mitochondrial (*Gpam*), Patatin-like phospholipase domain-containing protein 3 (*Pnpla3*), stearoyl-CoA desaturase-1 (*Scd1*), and fatty acid synthase (*Fasn*) (Figs. 7, 8A). GO term analysis indicated that all interacting genes were implicated in processes related to lipid homeostasis (Fig. 8C). On the other hand, only 2 DEGs, complement factor properdin (*Cfp*) and hemolytic complement (*Hc*)—related to complement/protein activation pathways, elevated in dystrophic animals, and downregulated by the additional lack of miR-378 (Fig. 7, Fig. 8D)—were revealed by STRING to interact with each other (Fig. 8E). Notably, IPA indicated activation of the complement system in *mdx* vs. WT counterparts (data not shown).

### Implications for metabolic alterations associated with by-product generation in *mdx* and *mdx*/miR-378^−***/***−^*** mice***

Additionally, we implemented the metabolic cages approach to examine potential changes in the total energy expenditure^48^. After a 3 day acclimation period, mice were kept in metabolic cages for an additional 24 h to assess food and water consumption, as well as feces and urine excretion. Though no differences in food intake were noticed (Supplementary Fig. 7A), the dystrophic animals displayed intensified defecation (Supplementary Fig. 7B) and *mdx*/miR-378^−/−^ mice showed increased water consumption (Supplementary Fig. 7C). Furthermore, it appeared that the urine volume excreted by the WT and dystrophic mice lacking miR-378^−/−^ was at a comparable level with significantly lower amounts in the *mdx* animals (Supplementary Fig. 7D). These effects were also associated with changes in urinary uric acid concentration, which was elevated in the dystrophic animals and diminished as a consequence of miR-378 loss (Supplementary Fig. 7E). Interestingly, based on the RNA-seq, IPA suggested increased levels of blood urea nitrogen and creatinine, indicators of kidney injury (Supplementary Fig. 7F). All of the above strengthens multi-organ perturbations in dystrophic animals, modeling human pathology which is not solely restricted to muscle tissues.

## Discussion

In this study, we comprehensively described the secondary consequences of dystrophin loss associated with alterations in systemic metabolism and we proposed the inhibition of miR-378 as a novel approach to mitigating those abnormalities, at least partially.

We functionally proved disturbed systemic carbohydrate metabolism by demonstrating a delayed glucose clearance from the bloodstream after a GTT and a completely blunted response following insulin administration in ITT in *mdx* mice, constituting the most commonly utilized model in DMD research. To the best of our knowledge, impaired insulin sensitivity in dystrophic males has not yet been reported, although insulin resistance has been revealed in female *mdx* mice^49^. Similar observations to our results regarding GTT in male dystrophic mice have been published by Stapleton et al.^9^ and Swiderski et al.^50^, although contradictory reports also exist^10,51^. It is known that the status of carbohydrate homeostasis may largely depend on the disease stage, as impaired glucose tolerance was observed only in severely immobilized DMD patients^13^, though the molecular mechanisms responsible for such abnormalities are not well understood. The vast majority of studies focused on skeletal muscles as the major players in glucose transport and homeostasis^52^. In our analysis, the protein level of GLUT4, a pivotal glucose transporter, was not affected in the dystrophic gastrocnemius muscle. However, Rodríguez-Cruz et al.^11^ found that GLUT4 forms cytoplasmic aggregates in muscle biopsies from DMD patients, which we also noticed in *mdx* mice, suggesting diminished glucose incorporation into the muscles. Moreover, altered insulin receptor affinity^13,53^ and GCs treatment^54^ were thought to trigger those complications. On the other hand, impaired insulin sensitivity, independent of GCs administration, was also observed in DMD boys^11^ and by us in non-treated *mdx* mice, pointing toward rather intrinsic causes of carbohydrate abnormalities in DMD. Accordingly, it appears that alterations in the dystrophin-glycoprotein complex (DGC) may facilitate the development of insulin resistance due to the impaired assembly of insulin receptors via DGC components^55^. DGC also binds neuronal nitric oxide synthase (nNOS), which facilitates glucose uptake into the muscles^56^. Thus, it might be suspected that the reduction of the nNOS pool in the absence of dystrophin contributes, at least partially, to the dysfunctional carbohydrate metabolism in DMD^57^. Importantly, apart from changes within dystrophic muscles, we and others^9^ found lower glycogen content and altered expression of genes implicated in glucose homeostasis in the liver. In the latter case, delta 4-desaturase, sphingolipid 1 (*Degs1*)—the most significantly upregulated factor in *mdx* livers compared with WT livers (*p*_adj_ = 5.68 × 10^–171^, fold change = 30.51)—could be proposed as one of the key players of the effects observed in dystrophic animals. Its deletion in the liver was reported to resolve insulin resistance in mice caused by leptin deficiency or an obesogenic diet^58^, among other effects.

Likewise, we provided some important insight into abnormalities in lipid homeostasis mediators which could be important for the development of dyslipidemia, recently described in DMD patients and animal models of the disease^59^. In the livers of *mdx* mice, we observed a dysregulated expression of genes affecting the level of circulating triglycerides, cholesterol, LDL, and HDL—such as *Apoa1*, *Abca1*, *Lpl* (normally not expressed in the liver under physiological conditions), *Pltp*, and *Pparg*^60^. Nonetheless, despite this molecular signature, we did not notice any significant abnormalities in the levels of triglycerides, total cholesterol, or HDL in the serum, under either fed or fasting conditions. No pathological accumulation of lipids in the liver was found either, in contrast to Murphy et al.^32^, who observed hepatic fat deposition in older, 6-month-old *mdx*-4Cv animals. On the other hand, Milad et al.^61^ did not find differences in the levels of total cholesterol, HDL, and triglycerides in the same model of the disease, namely 7-month-old *mdx*-4Cv mice fed a normal chow diet. However, because White et al.^59^ suggested that the elevation of lipids in DMD dogs progresses with age, it would be of particular importance for future studies to further investigate dyslipidemia features in older dystrophic mice, not only under normal conditions but also when animals are kept on an obesogenic diet. Importantly, lipid-lowering agents such as statins, have already been proposed to exert beneficial effects in DMD^62,63^; however, our^64^ and other studies^65^ did not confirm the rationale to utilize simvastatin, at least, to ameliorate muscle-related DMD symptoms. Our study indicated reduced hepatic expression of the key lipogenic factors, *Scd1* and *Fasn*, similarly to the results obtained in skeletal muscles of *mdx* animals^66^. In the latter study, Paran et al. reported that a reduction of approx. 50% in de novo lipogenesis contributes to the dysfunction of sarco/endoplasmic reticulum Ca^2+^-ATPase (SERCA) activity, whereas the recovery of this pathway through the overexpression of lipogenic factors mitigates SERCA function by affecting the composition of the sarcoplasmic reticulum membrane^66^. Of note, pharmacological activation of SERCA was recently demonstrated to provide a promising therapeutic strategy for DMD^67^. Moreover, apart from lipogenic genes, we observed decreased expression of the gene encoding lipid droplet coat protein perilipin 2 (*Plin2*) in dystrophic livers, similarly to the findings reported by Murphy et al.^32^ on protein level, further emphasizing the perturbations in lipid homeostasis displayed by dystrophic animals.

Finally, the most important finding is that we identified miR-378 as a factor that regulates not only muscle-related dysfunctions, as we showed previously^23^, but also metabolic disturbances in the *mdx* model of DMD, as reported in the current study. Though miR-378 is a well-documented mediator of metabolism^18^, this is the first report on its relevance to metabolic alterations under dystrophic conditions. *Mdx* mice devoid of miR-378 exhibited improved glucose tolerance, which is in line with studies performed not only in miR-378 knockout^25, 26,68^ but also in miR-378 overexpressing animals, which manifest impaired glucose and/or insulin tolerance^26,27,69^.

At the same time, we did not observe higher levels of the verified targets of miR-378, p110α^26^ and IGF1R in the livers of dystrophic mice lacking miR-378. This indicates that the molecular outcome of miR-378 loss might largely depend on the pathological conditions and the existence of factors that mask miR-378-specific effects. The analysis of potential mediators in the whole tissue is also associated with limitations, as the relevance of cell type-specific alterations might be overlooked in such experimental settings. On the other hand, we unravel the restoration of hepatic glycogen content in dystrophic mice devoid of miR-378. Importantly, in agreement with our findings, Liu et al.^26^ reported diminished glycogen content in the livers of miR-378 overexpressing mice.

miR-378 loss restored the expression of genes implicated in lipogenesis and lipid storage, such as *Fasn* and *Scd1,* which were diminished in the dystrophic mice. Of note, they were already reported to have higher levels in miR-378 knockout mice^26^, whereas *Scd1* was additionally verified as a direct target of miR-378^69^. Interestingly, bioinformatics analysis predicted intensified and decreased lipolysis in *mdx* and *mdx*/miR-378^−/−^ mice, respectively. We link this with the pattern of hepatic triglyceride content after a fasting period. However, no accumulation of triglycerides in *mdx* livers under food deprivation could be also explained by reduced lipogenesis, whereas their elevation in *mdx*/miR-378^−/−^ animals would reflect restored lipogenesis. In line with such an assumption, we propose that *Gpam,* a factor involved in the glycerophosphate pathway of de novo triglyceride synthesis in mammals, might serve as an important player in the observed effects. Although further studies are warranted, it was reported that knockout of *Gpam* results in a profound reduction of triglyceride biosynthesis^70^, among other things.

Lastly, PPARα was validated as a target of miR-378^68^; therefore, the drastic reduction of PPARα levels in the *mdx*/miR-378^−/−^ mice observed in the current study is likely the result of indirect regulation of this transcription factor by miR-378 loss under dystrophic conditions. We previously made similar findings concerning fibroblast growth factor 1 (FGF1), a predicted target of miR-378, which was significantly lower in *mdx*/miR-378^−/−^ than *mdx* mice^23^. Although the role of PPARα in DMD is poorly understood, its agonist, fenofibrate, is known to exert beneficial effects in *mdx* mice related to muscle pathology and fatty acid metabolism^71^. Moreover, PPARα inducers were proposed as factors that prevent insulin resistance^72^, but other results showed that PPARα knockout mice were protected from insulin resistance induced by a high-fat diet^73^. In light of the above, the use of PPARα antagonist would be important to resolve its potential to mimic the effect of miR-378 loss in dystrophic animals. Also, the evaluation of the potential link between PPARα, increased liver mass, and cardiac complications^74,75^ in dystrophic mice seems to be an interesting aspect of further studies.

In conclusion, we provided an insightful report on metabolic disturbances accompanied by the global, hepatic-specific changes displayed by the *mdx* model of DMD. Importantly, we uncovered for the first time that the lack of miR-378 in dystrophic mice can, at least partially, reverse those alterations. Although more mechanistic studies are warranted, we believe that the downregulation of miR-378 might serve as a promising therapeutic approach to mitigate the multifaceted symptoms of DMD.

## Materials and methods

### Animals

Animal procedures were performed after approval by the 2nd Institutional Animal Care and Use Committee (IACUC) in Kraków, Poland (approval numbers: 230/2018, 200/2019, 237/2019), following Polish and European legislation and according to the ARRIVE guidelines. Mice were housed under specific pathogen-free (SPF) conditions in individually ventilated cages with a 14 h/10 h light/dark cycle and were kept on a normal, chow diet with water and food available ad libitum (fed conditions). Under fasting conditions, mice were placed in a new cage with no food for 4 (in the morning) or 16 (overnight) hours, water ad libitum. Control (C57BL/10ScSnJ) and *mdx* (C57BL/10ScSn-*Dmd*^*mdx*^/J) mice were purchased from Jackson Laboratory (Bar Harbor, ME, USA, stock nos. 000476 and 001,801, respectively), whereas miR-378^−/−^ mice (129SvEv/C57BL/6) were kindly provided by Dr. Eric Olson (Department of Molecular Biology, University of Texas Southwestern Medical Center, Dallas, TX, USA)^25^. Mice globally lacking both dystrophin and miR-378 (*mdx*/miR-378^−/−^) were generated as described previously^23^. Wild-type (WT), *mdx*, and *mdx*/miR-378^−/−^ mice were bred at mixed C57BL/10ScSn x 129SvEv/C57BL/6 background^23^. For all experiments only male, 3-month-old animals were used.

### Tolerance tests

GTT and ITT were conducted according to the published protocols with minor modifications^76^. Briefly, GTT and ITT were performed in mice after an overnight (16 h) or 4 h fasting period, respectively. At first, fasting blood glucose was measured (indicated as 0 min timepoint) followed by intraperitoneal (i.p.) administration of 20% D-( +)-Glucose solution (Sigma-Aldrich, St. Louis, MO, USA) of a final dose of 2 g/kg body weight (BW) (GTT) or 1 U/mL insulin (Humulin® R, Lilly, Warsaw, Poland) in a dose of 0.5 U/kg BW (ITT). Next, the measurement of blood glucose concentration was repeated after 15, 30, 60, and 120 min (GTT) or after 15, 30, 60, and 90 min (ITT). Additionally, the random glucose level under non-fasting conditions (random glucose) and insulin under both non-fasting (random insulin) and after overnight (16 h) fasting conditions were measured. In any case, the blood glucose level was estimated from the tail tip using a glucometer (Diagnosis S.A., Białystok, Poland), whereas insulin level was assessed by Ultra Sensitive Mouse Insulin ELISA Kit (Crystal Chem, Elk Grove Village, IL, USA) in serum prepared from the blood collected from *vena cava*, after allowing the blood to clot at room temperature for 30 min and centrifugation at 2000 × *g* for 10 min at 4 °C.

### Metabolic cages

For the assessment of total energy expenditure, we utilized metabolic cages. Mice were housed individually in cages and acclimatized to standard animal house conditions for 3 days. Water and food were precisely measured to enable the determination of the food and water intake, feces, and urine excretion after consecutive 24 h. Upon collection, feces were dried for 1 h at 37 °C, whereas urine was centrifuged (2000 × *g* for 10 min at 4 °C). All results were calculated per kg BW.

### RNA isolation, reverse transcription, and quantitative real-time PCR (qRT-PCR)

For RNA isolation, tissues (a piece of a right lobe of the liver and gastrocnemius muscle) were collected, preserved in RNAlater solution (Sigma-Aldrich, St. Louis, MO, USA), snap-frozen in liquid nitrogen, and stored at −80 °C. Tissues were homogenized using TissueLyser in 1 mL of QIAzol Lysis Reagent (all from QIAGEN, Hilden, Germany) followed by RNA isolation that was performed according to our previous expertise using the standard Chomczynski-Sacchi method^77^. For the reverse transcription, 1 µg of total RNA was transcribed to cDNA in a mix containing recombinant M-MuLV reverse transcriptase (Thermo Fisher Scientific, Waltham, MA, USA), dNTPs, oligodT (both from Genomed, Warsaw, Poland), and water, or was conducted according to the miRCURY LNA RT Kit (QIAGEN, Hilden, Germany) in the case of miRNAs expression analysis. qRT-PCR was performed using StepOne Plus Real-Time PCR (Applied Biosystems—Thermo Fisher Scientific, Waltham, MA, USA) on obtained cDNA with SYBR Green PCR Master Mix, and specific primers (all from Sigma-Aldrich, St. Louis, MO, USA) which sequences are listed in Table 1. Eukaryotic elongation factor 2 (*Eef2*), with a stable expression between analyzed groups, served as a housekeeping gene. In the case of miRNA level assessment, qRT-PCR was conducted according to the miRCURY LNA SYBR PCR Kit with specific primers (miRCURY LNA™ miRNA PCR Assays, all from QIAGEN, Hilden, Germany). Normalization of miRNAs expression was done based on the small nucleolar RNA, C/D box 68 (*Snord68*) which level was not affected by mice genotype. Relative gene expression was calculated based on the comparative C_t_ method according to the 2^−ΔCt^ formula, where (ΔC_t_ = C_t gene of interest_ − C_t *Eef2/Snord68*_). Primer specificity was monitored based on the melting curves.

**Table 1:** The sequences of forward (F) and reverse (R) primers used in qRT-PCR.

Gene	Sequence 5′–3′
*Acly*	F:CTCCAAGAAGCCAAATCTTATC R:ATATTCATCAGCTTCCTCCC
*Agpat9*	F:CAGAAGGTACTTGCATCAAC R:GAACTGGGGGTTATACTTTATG
*Cfp*	F:TCGACACTGCTATAACATCC R:GAAGGTAACATTCTTCTCACC
*Eef2*	F:AGAACATATTATTGCTGGCG R:AACAGGGTCAGATTTCTTG
*Fasn*	F:GATTCAGGGAGTGGATATTG R:CATTCAGAATCGTGGCATAG
*Glut4*	F:GTAACTTCATTGTCGGCATGG R:AGCTGAGATCTGGTCAAACG
*Gpam*	F:CATTCAGATTCACAAGGGTC R:GTGAATCAAGGTACTGAAGAC
*Hc*	F:ATCTTCAGGTGGATTTTCAG R:CTCGAGTGAATCTTTAACCTG
*Plin2*	F:ATAAGCTCTATGTCTCGTGG R:GCCTGATCTTGAATGTTCTG
*Pnpla3*	F:GTGAATATCACCAACCTCAG R:TTACAGATGCCATTCTCCTC
*Ppara*	F:ACTACGGAGTTCACGCATGTG R:TTGTCGTACACCAGCTTCAGC
*Scd1*	F:GTGGGGTAATTATTTGTGACC R:TTTTTCCCAGACAGTACAAC

### Liver transcriptome sequencing

For RNA-seq, mice were euthanized, perfused immediately with saline containing 0.5 U/ml heparin (Polfa, Warsaw, Poland) through the left ventricle, and a piece of the liver (left lateral lobe) was collected to tubes containing RNAlater (Sigma-Aldrich, St. Louis, MO, USA). Samples were immediately snap-frozen in liquid nitrogen and stored at -80 °C before RNA isolation which was performed as described above according to the standard Chomczynski-Sacchi method^78^.

High-throughput gene expression profiling was conducted using next-generation sequencing and a highly multiplexed amplification method provided by Ion AmpliSeq™ technology and Ion Proton™ machine (Thermo Fisher Scientific, Waltham, MA, USA). Libraries for 16 samples were prepared using Ion AmpliSeq™ Transcriptome Mouse Gene Expression Kit covering > 20,000 mouse RefSeq genes in a single assay. Before the library preparation step, integrity and concentrations of RNA samples were determined using an Agilent 2100 Bioanalyzer with RNA 6000 Nano Kit (Agilent, Santa Clara, CA, USA). Libraries were prepared according to the manufacturer's protocol using 100 ng of total RNA as the input material. Emulsion PCR, templating and chip loading was conducted manually. Sequencing was performed using Ion PI Hi-Q Sequencing 200 chemistry. Two Ion PI Chip v3 chips were used with 8 barcoded and pooled in equimolar concentrations samples sequenced per single chip. The primary bioinformatics analyses including mapping were carried out using Torrent Suite Server v5.10.0. Transcripts were counted with the HTSeq Python package (https://htseq.readthedocs.io/en/release_0.10.0/) while read-count normalization and differential gene expression analysis (DGE) was carried out using DESeq2 package implemented in R version 3.3.3 software. *P*-values for differentially expressed genes were corrected for multiple comparisons using the Benjamini–Hochberg approach and the results with the corrected *P*-adjusted < 0.05 were considered significant if not otherwise stated. Heatmaps were generated using ggplot2 R package^79^ or were created in GraphPad Prism 8 Software. Gene set enrichment analysis (GSEA) was conducted with the GSEA method using the Java GSEA implementation. GSEA pre-ranked module and log_2_ fold change values were used as the input values (http://software.broadinstitute.org/gsea/index.jsp). Mapping to Gene Ontology terms (GO) was conducted using the “GOstats” package implemented in R version 3.3.3 software^80^. Data were analyzed through the use of IPA (QIAGEN, Hidden, Germany, https://www.QIAGENbioinformatics.com/products/ingenuitypathway-analysis)^81^ and STRING database for known and predicted interactions^82^. miRmap database with the default parameters was recruited to predict miR-378 targets^47^.

### Biochemical assays

The measurements of ALT, AST, total cholesterol, HDL, triglycerides, and bilirubin level in serum were performed using biochemical analyzer SPOTCHEM EZ SP-4430 (ARKRAY, Kyoto, Japan) according to the vendor’s instructions. Triglyceride content in the liver was assessed by Triglyceride Quantification Colorimetric/Fluorometric Kit (Sigma-Aldrich, St. Louis, MO, USA), whereas glycogen level was analyzed using Glycogen Colorimetric/Fluorometric Assay Kit (BioVision, Milpitas, CA, USA). The urinary uric acid concentration was tested according to the vendor’s instruction (CORMAY, Warsaw, Poland).

### Protein isolation

For protein isolation, collected tissues were homogenized using TissueLyser (QIAGEN, Hilden, Germany) in PBS containing 1% Triton X-100 (BioShop, Burlington, ON, Canada) and protease inhibitors (cOmplete™, Mini Protease Inhibitor Cocktail; Roche Diagnostic, Basel, Switzerland). After centrifugation (14,000 × *g* for 10 min at 4 °C), supernatants were collected and total protein content was assessed by the bicinchoninic acid assay (BCA) method (Sigma-Aldrich, Saint Louis, MO, USA).

### Western Blot

For the assessment of protein level of pAKT, AKT, p110α, GSK-3β, PPARα, GLUT4, GAPDH, and VINCULIN, 25 µg of protein lysates were subjected to SDS-PAGE electrophoresis, transferred to nitrocellulose membrane (wet transfer, 100 V/1.5 h, 4 °C), blocked in 5% non-fat milk (in the case of p110α, GSK-3β, PPARα, GLUT4, GAPDH) or BSA (for pAKT, AKT, and VINCULIN) in TBS containing 0.1% Tween20 (1 h, room temperature), and incubated overnight with primary antibodies diluted in the appropriate blocking solution (Table 2). If needed, the membranes were cut before the incubation with the desired antibody. The next day, membranes were washed, incubated with secondary antibodies (diluted in the appropriate blocking solution) conjugated to horseradish peroxidase (HRP) (Table 2) for 1 h at room temperature, washed again, and the chemiluminescence was measured by ChemiDoc™ MP Imaging System (Bio-Rad, Hercules, CA, USA) after addition of the Immobilon® Western Chemiluminescent HRP Substrate (Millipore, Burlington, MA, USA). For stripping and reprobing, the membranes were washed thoroughly with TBS containing 0.1% Tween20 followed by the incubation with 0.5 M glycine solution (pH = 2.6), twice for 30 min with washing steps after each incubation (3 times per 5 min). Afterward, the membrane was blocked and subsequent steps were done as described above. Densitometric analysis was performed with the use of ImageJ software. Unedited Western Blot results from all experiments subjected to densitometric analysis are presented in Supplementary Fig. 8.

**Table 2:** The list of antibodies used in Western Blot analysis.

I° antibody/dilution	Vendor/Cat. No	II° antibody/dilution	Vendor/Cat. No
Rabbit anti-mouse pAKT (Ser 473)/1:1000	Cell Signaling Technology, Danvers, MA, USA/9271S	Goat anti-rabbit/1:10000	Cell Signaling Technology, Danvers, MA, USA/7074
Rabbit anti-mouse AKT/1:1000	Cell Signaling Technology, Danvers, MA, USA/9272S	Goat anti-rabbit/1:10000	Cell Signaling Technology, Danvers, MA, USA/7074
Mouse anti-porcine GAPDH/1:2000	Santa Cruz Biotechnology, Dallas TX, USA/sc-59540	Goat anti-mouse/1:20000	BD Pharmingen, San Diego, CA, USA/554002
Rabbit anti-rat GLUT4/1:1000	Abcam, Cambridge, UK/ab654	Goat anti-rabbit/1:10000	Cell Signaling Technology, Danvers, MA, USA/7074
Rabbit anti-human GSK-3β/1:500	Santa Cruz Biotechnology, Dallas TX, USA/sc-9166	Goat anti-rabbit/1:5000	Cell Signaling Technology, Danvers, MA, USA/7074
Goat anti-human PPARα/1:500	Santa Cruz Biotechnology, Dallas TX, USA/sc-1985	Donkey anti-goat/1:5000	Thermo Fisher Scientific, Waltham, MA, USA/PA1-28664
Rabbit anti-human p110α/1:500	Cell Signaling Technology, Danvers, MA, USA/4249T	Goat anti-rabbit/1:5000	Cell Signaling Technology, Danvers, MA, USA/7074
Mouse anti-human VINCULIN/1:400	Sigma-Aldrich, St. Louis, MO, USA/ V9131	Goat anti-mouse/1:10000	BD Pharmingen, San Diego, CA, USA/554002

### ELISA

IL6, TNFα, TNFR1, and IGF1R levels in the liver were assessed by ELISA assay (R&D, Minneapolis, MN, USA, and Abbexa, Cambridge, UK, respectively) according to the vendor’s instruction using 100 µg of protein lysates as determined by the BCA method. The results were calculated per mg of the total protein.

### Histological analyses

A piece of the liver (right lobe) was collected directly to 10% formalin in PBS for 48 h. Afterward, tissues were transferred to 70% ethanol and stored at 4 °C before further processing. Tissues were dehydrated, embedded in paraffin, and cut using a microtome (Thermo Fisher Scientific, Waltham, MA, USA) on 4.5 µm sections. After deparaffinization, sections were subjected to hematoxylin and eosin (H&E) and Masson’s Trichrome staining according to the vendor’s instructions (Sigma-Aldrich, St. Louis, MO, USA). The pictures were taken using the Leica DMi8 microscope with the CMOS Leica MC170 HD camera. The assessment of liver injury was conducted according to previous reports^83^ with modifications. Briefly, semiquantitative total scoring of necrosis, vacuolization, congestion, and leukocyte infiltration was performed, based on the following scale (for each parameter): 0—none, 1—minimal, 2—mild, 3—moderate, 4—severe. The liver fibrosis was calculated using ImageJ software and presented as the percentage of Masson’s trichrome positive area of the whole liver biopsy/scan^84^.

Oil Red O (ORO) staining for the determination of lipid accumulation was performed on 5 μm thick frozen sections, which were cut after embedding the liver in an OCT medium and freezing in an isopentane-cooled liquid nitrogen bath. The sections were fixed in formalin, briefly washed with running tap water, rinsed with 60% isopropanol, and stained for 15 min with freshly prepared ORO (ICN Biomedicals, Inc., USA) working solution (0.5% of the stock solution prepared in isopropanol was diluted 1.6 times in water). Afterward, the sections were rinsed with 60% isopropanol, distilled water, and mounted with an aqueous mounting medium. The pictures were taken using the Nikon Eclipse Ti microscope.

Immunofluorescent staining of GLUT4 was performed on frozen sections according to the protocol described previously^19^, with the use of rabbit anti-rat GLUT4 antibody (Abcam, Cambridge, UK, ab654, 1:200) and Alexa Fluor 488 donkey anti-rabbit IgG (H + L) secondary antibody (Thermo Fisher Scientific, Waltham, MA, USA, A21206, 1:500). The pictures were taken using the Leica DMi8 microscope with Leica DFC7000 GT fluorescent camera.

### Isolation of primary hepatocytes

Isolation of primary hepatocytes was performed according to the established protocol^85^ with a modified digestion step in which 200 U/mL of collagenase IV (Thermo Fisher Scientific, Waltham, MA, USA) was used. For RNA isolation, cells were seeded at a density of 2 × 10^5^ cells/well of 12-well plates. 24 h after isolation, the medium was removed, cells were washed with PBS, lysed with Fenozol (A&A Biotechnology, Gdańsk, Poland), and subjected to RNA isolation and miRNA level determination as described *in “RNA isolation, reverse transcription (RT), and quantitative real-time PCR (qRT-PCR)”* section of the manuscript.

### Seahorse analyses

Oxygen consumption rate (OCR) in primary hepatocytes was measured using Seahorse Bioscience XFe96 Analyzer (Agilent Technologies, Santa Clara, CA, USA) according to our protocol described previously^86, 87^. Briefly, 1 × 10^5^ of live hepatocytes (determined by the trypan blue staining) were plated into Seahorse XFe96-well plates directly after isolation. The next day, the medium was switched for low-buffered assay medium (8.3 g/L DMEM, 2 mM L-Glutamine, 1 mM sodium pyruvate, and 0.5% phenol red (all from Sigma-Aldrich, St. Louis, MO, USA ) at pH = 7.4, and the cells were incubated at 37 °C, 20% O_2_, without CO_2_ for ~ 1 h. OCR was assessed at a basal level and after consecutive injections of 1.5 µg/mL oligomycin, 0.4 µM FCCP, and 0.5 µM rotenone + antimycin A (all from Sigma-Aldrich, St. Louis, MO, USA). All parameters were optimized before the test. Basal respiration was calculated as the last rate measurement before oligomycin injection minus non-mitochondrial respiration (minimum rate measurement after antimycin A and rotenone injection). The results were normalized to the protein content using the BCA method.

### Statistical analysis

The results are presented as mean ± SEM. In the figure legends, the “n” number indicates biological replicates equal to the number of mice used in each experiment. Differences between groups were tested for statistical significance using one-way ANOVA with Tukey’s posthoc test for multiple comparisons or two-way ANOVA for repeated measures with Tukey’s posthoc tests for multiple comparisons. When indicated, the unpaired 2-tailed Student’s t-test was applied test for the comparison of two groups. *p* < 0.05 was considered significant, whereas *p* = 0.05–0.1 was described as a tendency. Grubb’s test was used to identify outliers. Graphs and statistical analyses were performed with the use of GraphPad Prism 8 Software.

## Supplementary Information

Supplementary Information.
