# Supplementary material for: miR-378 affects metabolic disturbances in the mdx model of Duchenne muscular dystrophy
Source: Sci Rep. 2022 Mar 10;12:3945. doi: 10.1038/s41598-022-07868-z (PMC8913680; doi:10.1038/s41598-022-07868-z)
Supplement: Supplementary file 1 — Supplementary Information. [file 41598_2022_7868_MOESM1_ESM.pdf]

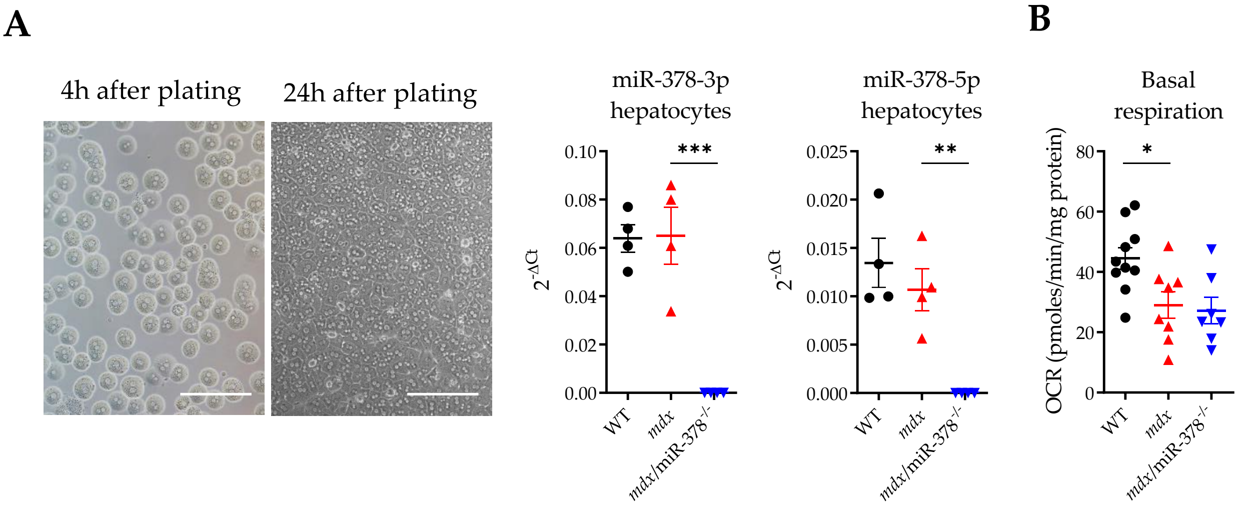

Supplementary Fig. 1. The level of miR-378 is not changed in hepatocytes from dystrophic mice and its lack does not further affect basal respiration. **(A)** Primary hepatocytes were isolated from the perfused livers; representative morphology shown on left together with the miR-378-3p and miR-378-5p levels assessed 24 hours after plating with the use of locked nucleic acid (LNA) technology; n=4/group. Scale bars represent 100 µm. **(B)** Basal respiration was determined in primary hepatocytes the next day after isolation utilizing Seahorse analyzer based on the oxygen consumption rate (OCR); n=7-10/group. Data are presented as mean ± SEM. *$p < 0.05$; **$p < 0.01$; ***$p < 0.001$ by one-way ANOVA with Tukey's post-hoc test.

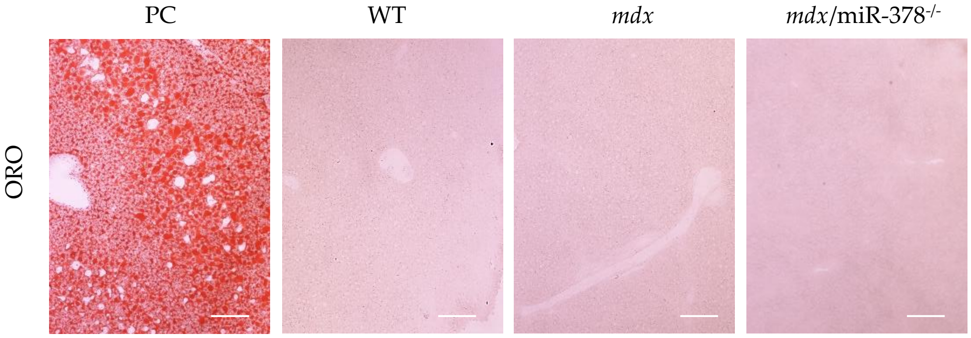

Supplementary Fig. 2. No accumulation of lipids is observed in the liver of WT, *mdx*, *mdx*/miR-378$^{-/-}$ mice. Lipid accumulation was assessed based on the Oil Red O (ORO) staining performed on liver frozen sections. Positive control (PC) was obtained from the mouse fed on a high-fat diet for 4 months. Scale bars represent 100 µm.

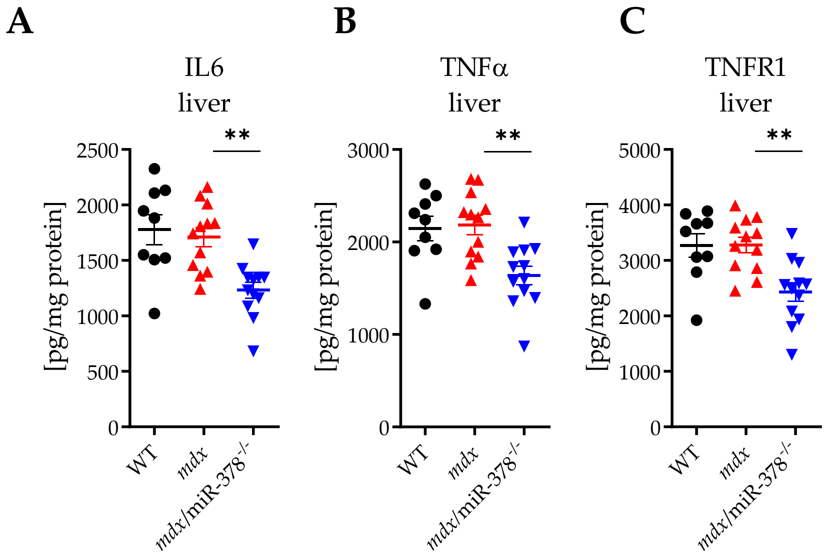

Supplementary Fig. 3. Interleukin 6 (IL6), tumor necrosis factor α (TNFα), and tumor necrosis factor receptor 1 (TNFR1) are decreased in the liver of dystrophic mice devoid of miR-378. The concentration of **(A)** IL6, **(B)** TNFα, and **(C)** TNFR1, measured by ELISA, n=9-12/group. Data are presented as mean ± SEM. $^{**}p < 0.01$ by one-way ANOVA with Tukey's post-hoc test.

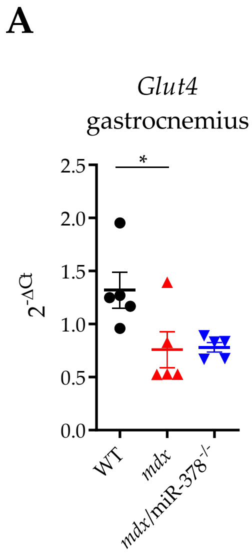

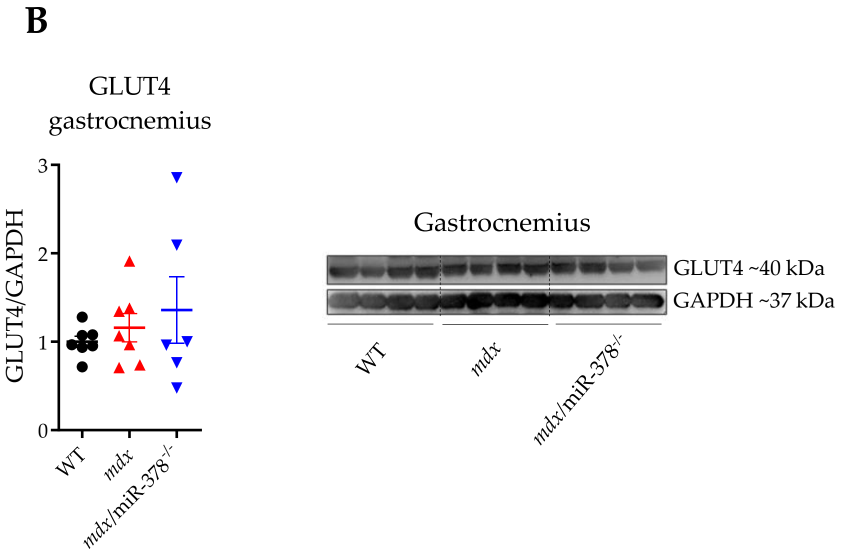

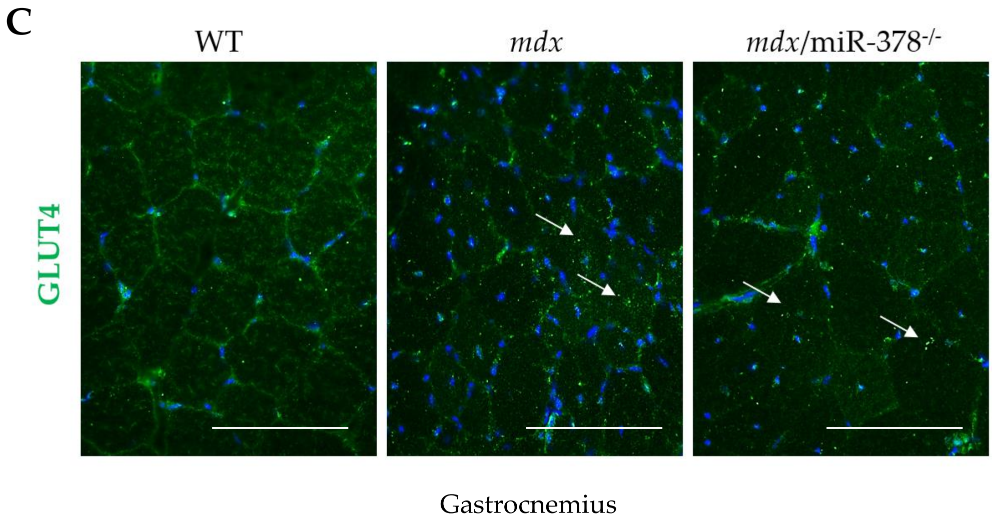

Supplementary Fig. 4. The analysis of glucose transporter 4 (GLUT4) level and localization in the gastrocnemius muscle. (A) *Glut4* mRNA was measured by qRT-PCR; n=5/group, whereas **(B)** GLUT4 protein was assessed by Western Blot together with densitometric analysis. Glyceraldehyde 3-phosphate dehydrogenase (GAPDH) served as loading control; n=6-7/group. **(C)** GLUT4 localization was assessed on frozen sections and visualized under the DMi8 microscope with Leica DFC7000 GT fluorescent camera. Arrows indicate exemplary cytoplasmic aggregates of GLUT4. n=5-6/group. Scale bars represent 100 µm. Data are presented as mean ± SEM. *$p < 0.05$ by one-way ANOVA with Tukey's post-hoc test.

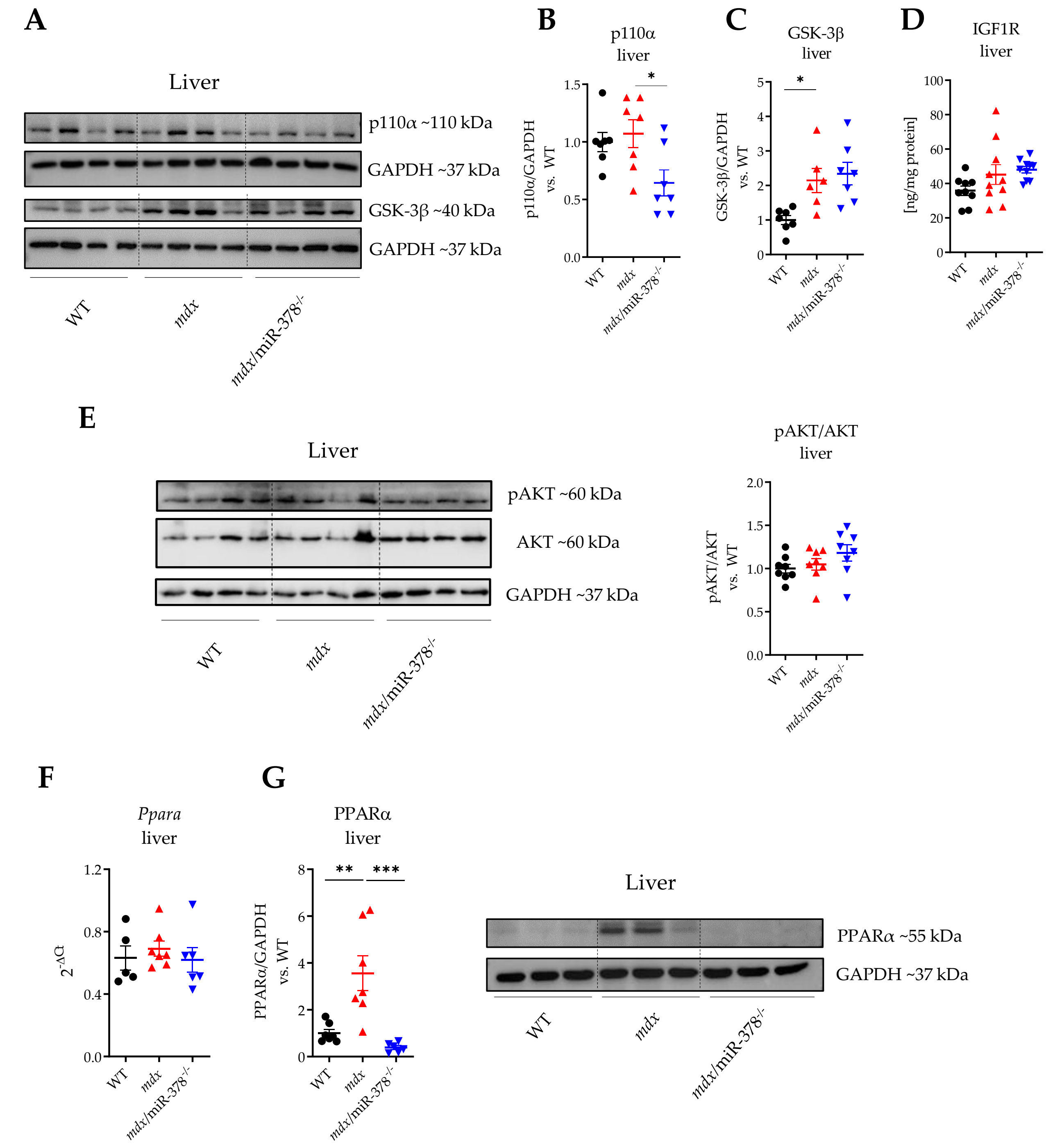

Supplementary Fig. 5. The analysis of carbohydrate and lipid metabolism mediators in the liver. (A) A representative result from Western Blot analysis of p110α subunit of PI3K and glycogen synthase kinase 3 beta (GSK-3β) together with densitometric analysis of **(B)** p110a and **(C)** GSK-3β. Glyceraldehyde 3-phosphate dehydrogenase (GAPDH) served as loading control; n=6-7/group. **(D)** Insulin-like growth factor 1 receptor (IGF1R) protein level in the liver assessed by ELISA, n=9-10/group. **(E)** A representative result from Western Blot analysis of pAKT (Ser 473) and AKT together with densitometric analysis of pAKT/AKT ratio. GAPDH served as a loading control, n=8/group. **(F)** mRNA level of peroxisome proliferator-activated receptor alpha (*Ppara*) determined by qRT-PCR; n=5-8/group, and **(G)** protein level of PPARα evaluated by Western Blot; densitometric analysis and representative Western Blot result, n=6-7/group. Data are presented as mean ± SEM. *$p < 0.05$; **$p < 0.01$; ***$p < 0.001$ by one-way ANOVA with Tukey's post-hoc test.

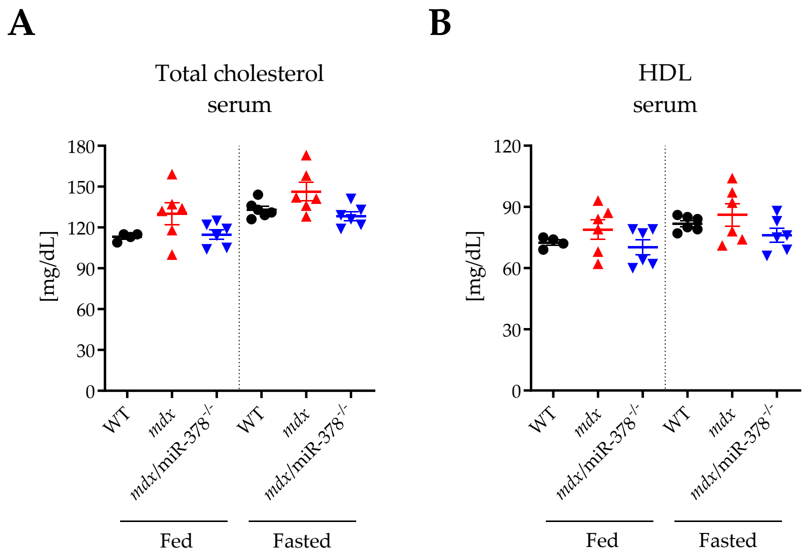

Supplementary Fig. 6. The level of **(A)** total cholesterol and **(B)** high-density lipoprotein-cholesterol (HDL) in the serum collected from mice upon non-fasting conditions (fed) and after overnight fasting (fasted); measurements performed with the use of biochemical analyzer SPOTCHEM; n=4-6/group. Data presented as mean ± SEM were tested for statistical significance with the use of one-way ANOVA with Tukey's post-hoc test.

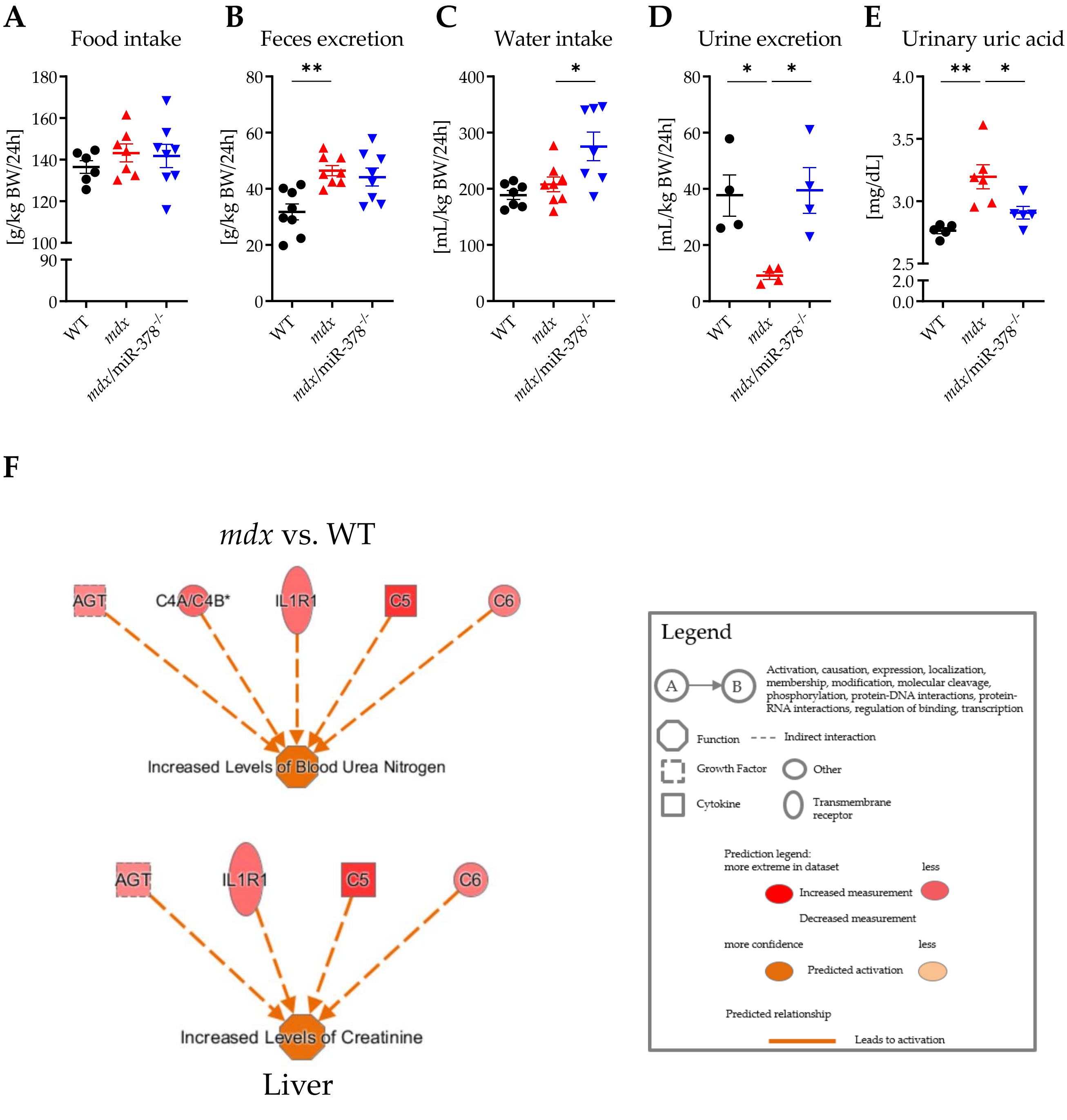

Supplementary Fig. 7. The investigation of total energy expenditure with the use of metabolic cages. **(A-D)** Mice were housed in individual metabolic cages. After acclimation time lasting 3 days, the **(A)** food intake; n=6-8/group, **(B)** feces excretion; n=8/group, **(C)** water intake; n=7-8/group, and **(D)** urine excretion; n=4/group, were assessed 24 hours later. The results were calculated per body weight (BW). **(E)** The uric acid concentration was examined in the urine collected after the metabolic cages experiment; n=5-6/group. **(F)** Ingenuity pathway analysis (IPA) predicted increased (indicated by red color) expression of factors associated with the level of blood urea nitrogen (p-value = 6.62×10^{-4}) and creatinine (p-value = 4.70×10^{-2}) in the liver of *mdx* vs. WT animals. Data are presented as mean ± SEM. *$p < 0.05$; **$p < 0.01$ by one-way ANOVA with Tukey's post-hoc test.

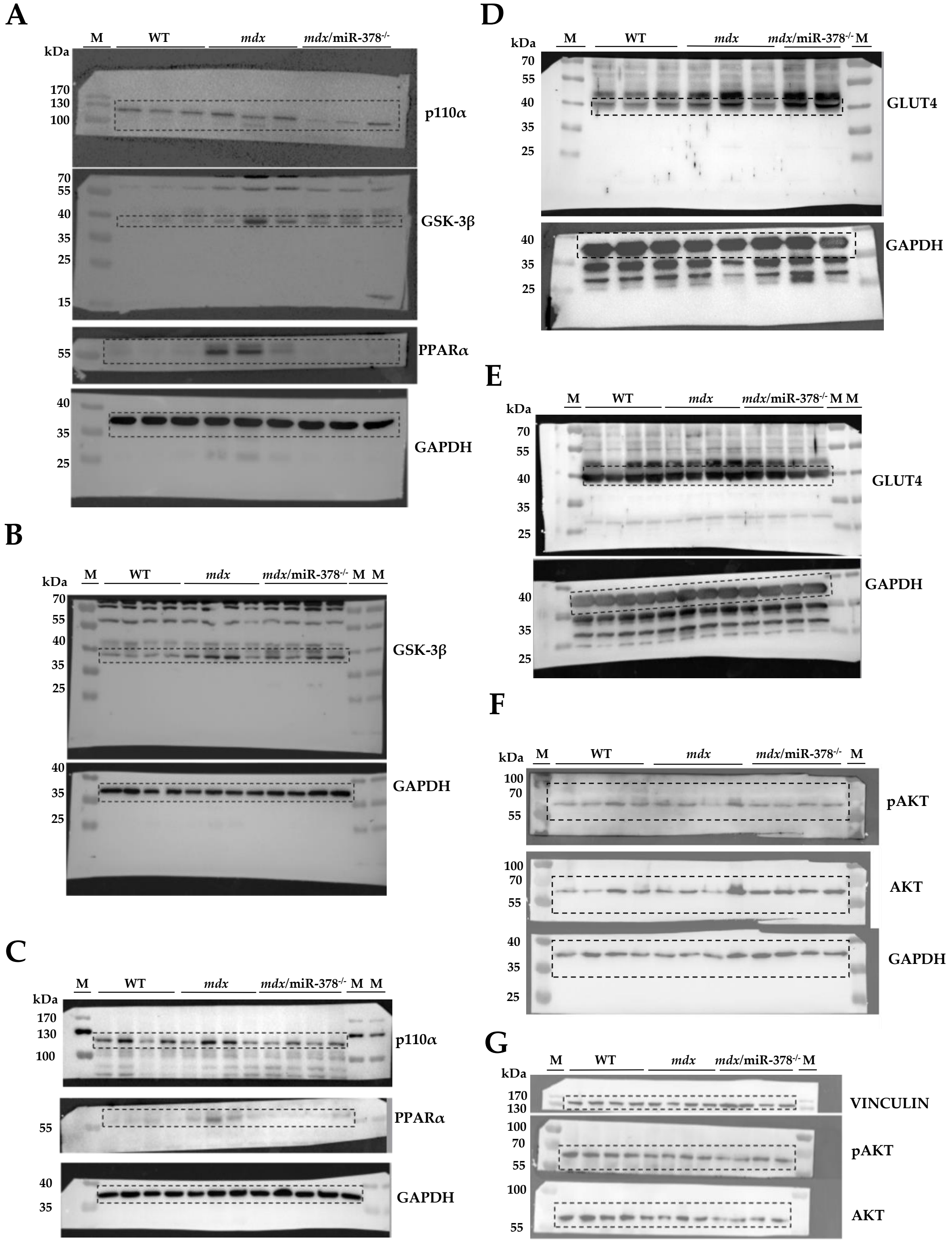

Supplementary Fig. 8. Unedited Western blot results. **(A-G)** Each panel represents a separate membrane with indicated proteins. Membranes were cut before incubation with desired antibodies and stripped if needed. Appropriate bands were chosen based on the predicted molecular weight. Unedited Western blot of (**A**) p110*α*, GSK-3β, and PPAR*α*; (**B**) GSK-3β; (**C**) p110*α* and PPAR*α*; (**D**,**E**) GLUT4; (**F**,**G**) pAKT and AKT. GAPDH or VINCULIN served as loading controls. For each analyzed protein, densitometric analysis of all bands marked in brackets (representing biological replicates equal to the number of animals used) was performed, followed by the statistical analysis and outlier identification by Grubb's test. The densitometric analysis of GLUT4 (**D, E**) is presented in Supplementary Fig. 4B together with representative Western blot (**E**). The densitometric analysis of p110*α* (**A, C**) is presented in Supplementary Fig. 5B, whereas representative Western blot (**C**) is shown in Supplementary Fig. 5A. The densitometric analysis of GSK-3β (**A, B**) is presented in Supplementary Fig. 5C, whereas representative Western blot (**B**) is shown in Supplementary Fig. 5A. The densitometric analysis of PPAR*α* (**A, C**) is presented in Supplementary Fig. 5G together with the representative Western blot (**A**). The densitometric analysis of pAKT and AKT (**F, G**) is presented in Supplementary Fig. 5E together with the representative Western blot (**F**). **M** – marker.
